# Phytotherapeutic Approaches to the Prevention of Age-Related Changes and the Extension of Active Longevity

**DOI:** 10.3390/molecules27072276

**Published:** 2022-03-31

**Authors:** Olga Babich, Viktoria Larina, Svetlana Ivanova, Andrei Tarasov, Maria Povydysh, Anastasiya Orlova, Jovana Strugar, Stanislav Sukhikh

**Affiliations:** 1 Institute of Living Systems, Immanuel Kant Baltic Federal University, A. Nevskogo Street 14, Kaliningrad 236016, Russia; olich.43@mail.ru (O.B.); surinac@mail.ru (V.L.); SSukhikh@kantiana.ru (S.S.); 2 Natural Nutraceutical Biotesting Laboratory, Kemerovo State University, Krasnaya Street 6, Kemerovo 650043, Russia; 3 Department of General Mathematics and Informatics, Kemerovo State University, Krasnaya Street 6, Kemerovo 650043, Russia; 4 Department of Pediatrics and Preventive Medicine, Medical Institute, Immanuel Kant Baltic Federal University, 14 A. Nevskogo ul., Kaliningrad 236016, Russia; drup1@yandex.ru; 5 Department of Biochemistry, Saint Petersburg State Chemical Pharmaceutical University, Professora Popova 14A, Saint Petersburg 197376, Russia; maria.povydysh@pharminnotech.com; 6 Department of Pharmacognosy, Saint Petersburg State Chemical Pharmaceutical University, Professora Popova 14A, Saint Petersburg 197376, Russia; lanas_95@mail.ru (A.O.); jovana.strugar12@gmail.com (J.S.); 7 Department of Science and Training of Scientific and Pedagogical Personnel, Saint Petersburg State Chemical Pharmaceutical University, Professora Popova 14A, Saint Petersburg 197376, Russia

**Keywords:** aging of the human body, medicinal plants, antioxidant, anti-inflammatory, anti-glycation, anti-neurodegenerative properties

## Abstract

Maintaining quality of life with an increase in life expectancy is considered one of the global problems of our time. This review explores the possibility of using natural plant compounds with antioxidant, anti-inflammatory, anti-glycation, and anti-neurodegenerative properties to slow down the onset of age-related changes. Age-related changes such as a decrease in mental abilities, the development of inflammatory processes, and increased risk of developing type 2 diabetes have a significant impact on maintaining quality of life. Herbal preparations can play an essential role in preventing and treating neurodegenerative diseases that accompany age-related changes, including Alzheimer’s and Parkinson’s diseases. Medicinal plants have known sedative, muscle relaxant, neuroprotective, nootropic, and antiparkinsonian properties. The secondary metabolites, mainly polyphenolic compounds, are valuable substances for the development of new anti-inflammatory and hypoglycemic agents. Understanding how mixtures of plants and their biologically active substances work together to achieve a specific biological effect can help develop targeted drugs to prevent diseases associated with aging and age-related changes. Understanding the mechanisms of the biological activity of plant complexes and mixtures determines the prospects for using metabolomic and biochemical methods to prolong active longevity.

## 1. Introduction

One of humanity’s global problems is the preservation of quality of life as the average age of the population rises. According to the 2019 Revision of World Population Prospects, by 2050, 1 in 6 people in the world will be over 65 (16% of the population), compared to 1 in 11 in 2019 (9% of the population) [1]. By 2050, one in four people in Europe and North America will be 65 years of age or older. In 2018, for the first time in history, the number of people aged 65 and over exceeded the number of children under the age of five worldwide. The number of people aged 80 and over is projected to triple, from 143 million in 2019 to 426 million in 2050 [1].

Chronic diseases were responsible for more than two-thirds of deaths worldwide (38 million) in 2014, according to the World Health Organization [2]. Most deaths from chronic diseases were associated with cancer, cardiovascular disease, chronic respiratory disease, or diabetes. Although chronic diseases have a major cumulative impact on human health and aging, epidemiology has historically studied chronic diseases separately [3]. Understanding the epidemiology of particular diseases requires a clinical assessment of each chronic disease that contributes to aging. Accounting for multiple chronic diseases at the same time, on the other hand, more accurately reflects the experience of patients who accumulate conditions associated with aging and better reflects overall health. Well-established data show that the incidence of cancer, cardiovascular disease, chronic respiratory disease, and diabetes is associated with common modifiable risk factors such as alcohol use, body mass index (BMI), smoking, unhealthy diet, and physical inactivity, which account for more than two-thirds of the diseases that cause human aging [4].

A significant increase in the proportion of the elderly in populations of developed countries has resulted in an increase in mortality from major diseases of old age (cardiovascular diseases, malignant neoplasms, neurodegenerative processes, decreased resistance to infection, diabetes mellitus) [5]. As a result, it is no coincidence that the concept of healthy aging is listed as one of the top priorities in the UN Program on Aging’s project “Programs for Scientific Research on Aging in the Twenty-First Century” [5].

Human aging is accompanied by the accumulation of unhealthy changes in the structure of cell biopolymers and the intercellular matrix. The age-related increase in the level of oxidative processes in cells and tissues is one of the most important factors influencing these changes [6]. With aging, assimilation processes in organs and tissues weaken, and the system of neurohumoral regulation of metabolism and body functions undergoes restructuring. Moreover, one of the leading reasons for this is systemic inflammation and the development of atherosclerotic processes. Oxidative stress associated with inflammation leads to significant changes in the structure of biomolecules. The rate of nonenzymatic protein modifications—oxidation, glycation, and lipooxidation, as well as amyloidogenesis—increases dramatically with age [7]. As a result, aging is often accompanied by the development of metabolic disorders (the most common of which is type 2 diabetes) as well as neurodegenerative diseases. In the process of aging, numerous changes of various natures accumulate in the human body. Therefore, it appears obvious that in order to effectively prolong active longevity, a coordinated effect on all of these factors associated with body aging is required. At the same time, we must not forget that neurodegenerative changes and diabetes mellitus complications are irreversible. Therefore, it is preferable to prevent them, for example, by using substances that prevent the development of these diseases with age—geroprotectors [8]. Clearly, a pharmaceutical strategy that aims to simultaneously prevent the full range of age-related molecular changes such as inflammation, oxidative stress, oxidative and glycoxidative protein modifications, and amyloidogenesis would be more effective than current approaches.

Over the past decades, natural compounds of plant origin have been intensively studied as potential antioxidants, antiglycators, and neuroprotective agents. However, their combined application remains largely intuitive [9]. One of the promising objects for preventing oxidative, inflammatory, neurodegenerative and glycating aging processes are the components of medicinal plants [9]. It has been shown that many secondary plant metabolites can effectively control the aging process and delay the development of age-related diseases [9].

Therefore, the goal of this research was to study the concept of using medicinal plants and their highly purified complexes, which have the optimal combination of antioxidant, anti-inflammatory, anti-glycating, and anti-neurodegenerative properties, to slow down the onset of age-related changes and prolong active longevity.

The scientific publications and patents of Russian and foreign authors on the effect of medicinal plants and herbal preparations on antioxidant activity, the ability to reduce the harmful effects of free radicals and, as a result, oxidative and aging processes in the human body, were the subjects of this study. Several keyword combinations were used to search PubMed for studies published between 1999 and 2022, including natural compounds, flavonoids, bioflavonoids, aging; medicinal plants; antioxidant, anti-inflammatory, anti-glycation, and anti-neurodegenerative properties. Abstracts, bibliographies, editorials, and pieces written in languages other than English and Russian were excluded. Generalization was the primary method [10]. The results of practical studies and original studies of the composition and antioxidant properties of medicinal ingredients, as well as statistical and clinical data on the antioxidant activity of plant ingredients (for example, plant extracts), scientific principles for the use of plant ingredients in the production of medicines, and the results of practical and original studies of the composition and antioxidant properties of medicinal ingredients were analyzed.

## 2. Antioxidant Properties of Medicinal Plants and Their Complexes

There is no doubt about the importance of disturbances in the regulation of free-radical processes that occur in the body during aging, which is one of the causes of severe pathologies such as atherosclerosis, myocardial infarction, diabetes, cancer, and a number of other diseases, the emergence and progression of which depend on the effect of unfavorable environmental factors, and in some cases, genetic anomalies. In living cells, there is a perfect system of antioxidant protection that regulates the formation of free radicals (FRs) and limits the accumulation in cells of both the FRs themselves and the toxic products of their activity. Accumulation of FRs in the body with aging increases due to a decrease in the effectiveness of the natural antioxidant system caused by exposure to radiation, UV radiation, smoking, alcohol, constant stress, and poor nutrition. Cells use a variety of body defense mechanisms against the toxic effects of free radicals. As a result, therapy with antioxidants (AO) is increasingly being used in the treatment of a variety of diseases. At the same time, the production of branded antioxidant preparations, which include various components of natural or synthetic origin, is expanding [11].

As previously stated [12], free radicals are atoms, molecules, or ions that have unpaired electrons and are extremely active in chemical reactions with other molecules. In a biological system, free radicals are often formed from oxygen, nitrogen, and sulfur molecules. These free radicals are members of the reactive oxygen species (ROS), reactive nitrogen species (RNS), and reactive sulfur species (RSS) groups of molecules. For example, ROS include free radicals such as superoxide anion, perhydroxyl radical, hydroxyl radical, nitric oxide, and other compounds such as hydrogen peroxide, singlet oxygen, hypochlorous acid, and peroxynitrite [13]. ROS are produced during cellular metabolism and functional activity and play an important role in cell signaling, apoptosis, gene expression, and ion transport [14]. However, excessive amounts of ROS can have harmful effects on many molecules, including proteins, lipids, RNA, and DNA, because they are very small and highly reactive. ROS can attack bases in nucleic acids, amino acid side chains in proteins, and double bonds in unsaturated fatty acids, in which the hydroxyl radical is the strongest oxidizing agent. ROS attacking macromolecules are often referred to as oxidative stress. Cells can usually protect themselves from ROS damage by using intracellular enzymes to maintain low ROS homeostasis. ROS levels can grow rapidly during periods of environmental stress and cellular malfunction, causing considerable cell damage in the body. Thus, oxidative stress makes a significant contribution to the pathogenesis of inflammatory diseases, cardiovascular diseases, cancer, diabetes, Alzheimer’s disease, and, in general, aging of the body [15]. The human body and other species have developed an antioxidant defense mechanism that comprises enzymatic, metal chelating, and scavenging activities to neutralize free radicals once they have been created in order to avoid or mitigate ROS-induced oxidative damage. Furthermore, plant antioxidants can aid in maintaining a healthy antioxidant level in the body [16].

The last twenty years have been marked by increased attention, both in medicine and the chemical industry, to the products of processing of medicinal plant raw materials (MPRM), which contain a rich complex of biologically active substances (BAS), many of which exhibit antioxidant activity [16,17]. The search, methods for isolation and study of promising natural sources of substances with antiradical activity (ARA) and antioxidant activity (AOA), along with the development of accessible and rapid methods for determining AOA, is currently one of the urgent tasks in preventing aging of the human body for modern medicine, pharmacy, cosmetology, and food industry.

**Figure 1:**
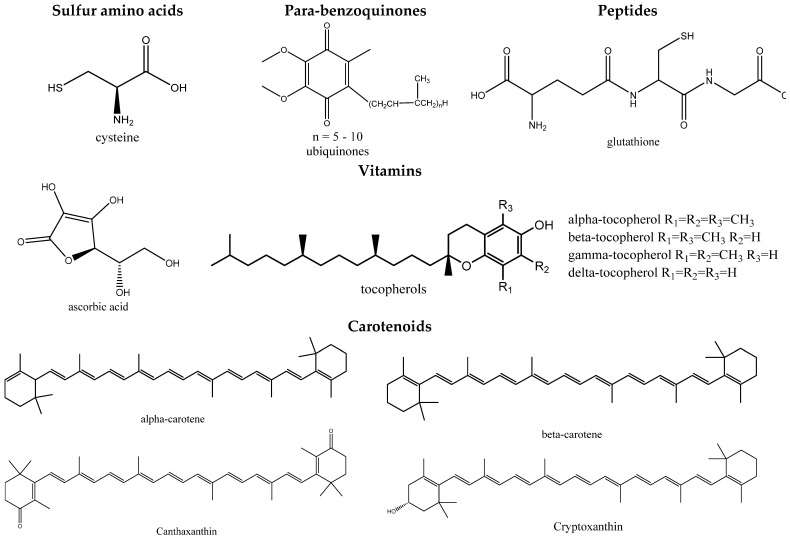
Structural formulas of some antioxidants.

**Figure 2:**
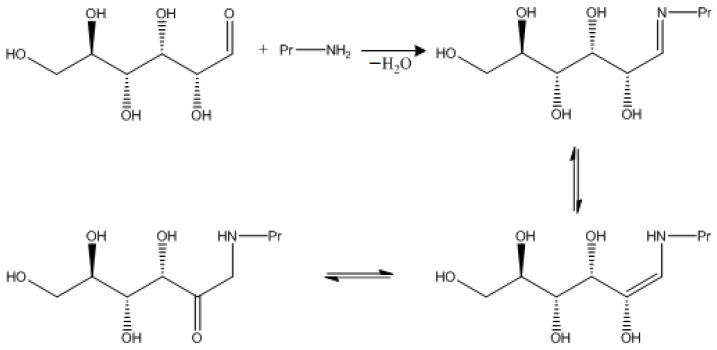
Maillard reaction scheme.

The group of substances that prevent the formation of strong oxidizing agents in vivo during the aging of the human body is diverse. These include the SH-containing amino acid cysteine, some peptides, and proteins (glutathione, albumin), ubiquinone, ascorbic and uric acids, tocopherols, carotenoids, flavonoids, etc. (Figure 1 and Figure 2). The detection of AOA makes it possible to judge the possible physiological value of the studied plant objects. Determining the content of individual AOs is, as a rule, insufficient since, in this case, the processes of mutual oxidation/reduction and the influence of the analyte matrix are not taken into account [18,19].

A significant amount of natural AO of the phenolic class present in MPRM determines their antioxidant effect. The content of flavonoids, along with ascorbic acid and provitamin A, is the most important indicator of the biological value of MPRM. The synergism of the action of ascorbic acid with flavonoids in the regulation of redox processes is significant [20]. The biological activity of natural AO is based on the processes of inhibition of the developing radical oxidation of tissue lipids through the interaction of active radicals with bioantioxidants [21]. It is noteworthy that the AOA value of flavonoids significantly decreases as the number of free phenolic hydroxyl groups in the molecules decreases: quercetin > rutin > luteolin-7-glucoside > apigenin > naringenin > 7-hydroxy-flavone [22]. For example, an assessment of the AOA of various natural flavonoids showed that quercetin and cyanidin have the highest AOA after theaflavin. Quercetin glycosides, such as rutin, have a lower AOA; flavones and flavone glycosides are characterized by the smallest AOA among this group of substances. Therefore, the antioxidant properties of plant raw materials can be judged based on the quantitative content of phenolic substances [20].

It has been established that polyphenols, tocopherols, and flavonoids exhibit AOA. The theory of radical oxidation distinguishes between the mechanisms of linear termination of radical chains on an inhibitor and the mechanism of inhibition, which is realized through the formation of complexes of active radicals with systems with conjugated π-bonds. For example, spatially shielded phenols, tocopherols, interact according to the first mechanism; these inhibitors have a distinct induction period. The second mechanism is realized more often for natural mixtures, including essential oils. Chamazulene and neryl methyl butanoates, two of the compounds found in them, may act as second-type inhibitors [23]. Antioxidants are widely used as the main means of therapy or as additional means of correction in the treatment of atherosclerosis, coronary heart disease, acute cerebrovascular accident, inflammatory processes, diabetes mellitus, a wide range of eye diseases, etc. [24].

Natural antioxidants used in pharmacy easily and organically enter into metabolic processes in the body during aging and practically do not have side effects common in synthetic drugs [25]. However, Henkel et al. [26] found that increased attention is paid to antioxidant therapy to prevent the rapid aging of the population. These substances are appealing because they are considered natural and are associated with a healthy diet. The hypothesis is that reducing oxidative stress can prevent disease processes such as cancer or coronary heart disease. Because the majority of the general population is comprised of reasonably healthy individuals, it is critical that these supplements be free of toxicity and side effects. While early research on antioxidant supplements suggested they could help prevent disease, more recent clinical trials and meta-analyses have cast doubt on their effectiveness. Several studies have shown that overconsumption of supplements can actually be harmful [27,28,29,30]. These studies have shown that excessive antioxidant levels can be teratogenic for embryos [31]. As a result, recent attention has been focused on adjusting the use of antioxidants for the treatment of male infertility, with particular emphasis on the potentially dangerous consequences of antioxidant therapy [26].

However, their composition is difficult to control [31]. The synergy of the action of complexes of plant and synthetic drugs may be the solution to this problem. Synergy is a process in which certain substances interact with each other to achieve a combined effect that is greater than the sum of their individual effects [32]. It can be considered as a natural direct strategy for increasing the efficacy of drugs with antioxidant activity. Hence, synergistic effects can be observed when plant preparations interact with conventional drugs or biochemical compounds. It is essential to identify and utilize these interactions because any advancement made as a result of such a process could be used to successfully treat human diseases. Even in diseases as complex as cancer or aging, favorable synergistic interactions between plants and medications must be investigated in order to get the optimum results, such as increased patient benefit or the avoidance of negative side effects. Multi-drug therapy is an effective strategy for directly blocking or destroying harmful agents (such as cancer cells, free radicals, or pathogens) while also activating the human body’s defense or repair mechanisms. This is because the previously accepted dogma of monodrug therapy has gradually been abandoned; for decades, pharmacological research has been based on the identification of a single active principle [33]. In terms of herbal medicine research, traditional Chinese medicine, Ayurveda, and traditional Western herbal medicine have only recently been scientifically validated and appreciated. Furthermore, over the last two decades, there has been an increase in the use of traditional medicines in combination with complementary and alternative medicine (CAM), which includes not only homeopathy, naturopathy, chiropractic, and energy medicine, but also ethnopharmacology and herbal medicine [34]. It is becoming clear that many diseases have a diverse etiology and can be treated more effectively with a combination drug strategy than with a single therapy. In Western countries, effective combination drug therapy is typically used for multifactorial or complex disease treatment (for example, cancer, hypertension, metabolic and inflammatory diseases, acquired immunodeficiency syndrome (AIDS), aging, oxidative and infectious processes) [35]. Herbal medicine and ethnopharmacology play an important role in the prevention of body aging in this context, as they are based on herbs or plants, which are secundum naturam, a complex pool of millions of molecules. It should be noted that human pharmacotherapy began with the use of plants in ancient times, probably mimicking the self-medication of animals.

Due to the huge popularity of CAM (including ethnopharmacology and herbal medicine), there is a need to focus on the risk/benefit ratio of herbal medicines and updated information. As a result, new information on synergistic anticancer and antioxidant effects of herbal medications and standard synthetic treatments is needed to explore the interaction of plants and drugs. In this regard, research into the complex of medicinal preparations with antioxidant properties is an urgent task.

The group of antioxidant protection of the human body from aging includes fat-soluble plant antioxidants: vitamins of group E (tocopherols), ubiquinone, vitamins of group A (retinols) and provitamins of group A (α-, β-, γ-carotenes), vitamins of group D (calciferols), K (phylloquinone and menaquinone), lipoic acid, etc. [36].

The mechanism of the antioxidant action of these compounds is due to their high donor properties (decrease in the amount of free oxygen in the cell, for example, by activating its utilization, increasing the activity of oxidation and phosphorylation processes), and the ability to restore lipid radicals. All of these compounds are classified as antiradical protection substances or direct antioxidants. Endogenous direct antioxidants are antioxidants that form less reactive radicals and have a more pronounced antioxidant activity. Thus, tocopherols are of the greatest importance among all known endogenous antioxidants [37]. However, the total antioxidant activity of these substances (the ability to inhibit peroxide free radical reactions at all stages of oxidative stress) is determined not only by their antiradical activity but also by the ability of the formed radical of the antioxidant itself, in parallel with recombination reactions with the formation of stable molecules, to initiate new chains of free radical oxidation upon interaction with each new molecule of an oxidized compound [38].

Endogenous direct plant antioxidants, which form less reactive radicals, have a more pronounced antioxidant activity. Thus, tocopherols are of the greatest importance among all known endogenous antioxidants. To date, seven different compounds that exhibit E-vitamin activity have been isolated from natural plant sources and studied. Phospholipids of mitochondria and endoplasmic reticulum of membranes have a specific affinity for α-tocopherols. The presence of a side isoprene chain in tocopherols, corresponding in length to the fatty acid residues of phospholipids, provides them with the ability to integrate into the membrane with subsequent formation of complexes between the methyl groups of the side chain and double bonds of fatty acids [39].

Plant α- and γ-tocopherols (vitamin E) have a pronounced antioxidant activity; the antiradical activity is higher for α-tocopherol and the antioxidant activity is higher for γ-tocopherol. α-Tocopherol provides 60% of the anti-radical action of all fat-soluble plant antioxidants. In addition to the antiradical action, α-tocopherol has the greatest ability to stabilize membranes and form complexes with fatty acids, leading to an increase in membrane resistance to free radicals [40]. Fat-soluble plant antioxidants are retinols and their precursors, mainly β-carotene [41].

The study of the pharmacological activity of *Calluna vulgaris* (L.) Hull., including as an antioxidant agent, is of undoubted interest. Since ancient times, this plant has been used in folk medicine for the treatment of various diseases: atherosclerosis, coronary heart disease, diabetes mellitus, and diseases of the musculoskeletal system. The chemical composition of the plant is quite diverse and includes the following groups of compounds: flavonoids (from 0.5% to 5.5%), catechins, proanthocyanidins (up to 7–8%), phenolic acids (from 5% to 9%), organic acids, amino acids (up to 17%), polysaccharides (about 5%), etc. [42]. It is known that the flavonoids of *Calluna vulgaris* have a pronounced antioxidant activity [43]. Inflammation in damaged tissues intensifies the formation of free radicals, the excess of which can adversely affect healthy cells and tissues. In this case, the use of antioxidants can reduce the risk of oxidative damage.

The selection of dosage form, including the justification of the extractant, plays an important role in the development of a drug. It is critical that the active substances be extracted as much as possible when preparing a dosage form such as an extract. During the extraction of *Calluna vulgaris* shoots with ethyl alcohol, mainly quercetin and its glycosides pass into the extraction. Chepel V. et al. [44] associated the antioxidant properties of *Calluna vulgaris* shoots with kaempferol-3-β-D-galactoside. This compound predominated in the ethyl acetate fraction of the ethanol extract from the aerial part of the plant. Previously, the *Calluna vulgaris* shoot extract was found to be safe for long-term use in an experiment on rats [45]. *Calluna vulgaris* was studied as a plant antioxidant in order to establish the nature of the dose–effect relationship for *Calluna vulgaris* shoot extract in an in vivo model, as well as to evaluate the antioxidant activity of its main component, quercetin-3-β-D-glucoside [42]. *Comarum palustre* L. has a wide spectrum of biological activity, including wound healing, analgesic, anti-inflammatory, immunostimulating, antirheumatic, and antioxidant actions [44]. *Comarum palustre* L. is widely used in traditional and folk medicine. The chemical composition of *Comarum palustre* L. is characterized by great diversity; it includes a polyphenolic complex, essential oils, resins, organic, hydroxycinnamic acids, and their derivatives [43], including chlorogenic acid.

Chlorogenic acid (CA)-3-O-caffeoylquinic acid and its isomers are powerful antioxidants. The properties of CA have been intensively studied over the past few years due to the discovery of a wide range of biological activities. CA exhibits the ability to inhibit tumor growth (in vitro), has an inhibitory effect on colorectal cancer, liver cancer, and laryngeal cancer, helps prevent type 2 diabetes mellitus, and has antihypertensive, antiviral, antibacterial, and antifungal effects [46]. At the same time, CA has relatively low toxicity and no side effects. Because of these properties, CA is used in food supplements and cosmetics with antioxidant properties. It has also been established that one of the molecular-cellular mechanisms of action of the *Comarum palustre* L. extract is its ability to inhibit the processes of free radical oxidation of biomacromolecules, probably due to the high content of substances of a phenolic nature. It has been shown that the mechanism of the antioxidant activity of this phytoextract is associated with its ability to increase the potential of the endogenous defense system of the body [47].

*Artemisia vulgáris* L. has been used as a medicinal plant since ancient times. It is also used in modern scientific medicine. In modern folk medicine of the countries of Central Asia, the herb *Artemísia vulgáris* L. in the form of a decoction is used as a laxative, diaphoretic, diuretic, and anthelmintic, is used as a sedative and anticonvulsant in Chinese medicine, and in Russian folk medicine in the treatment of bronchial asthma, bleeding, pyoderma, and as an antitoxic, antioxidant, and tonic. Extracts of *Artemisia vulgaris* L. have a cytotoxic effect on leukemia cells. Their choleretic, hypoglycemic, antioxidant, and hypolipidemic properties have also been determined [48]. *Artemisia vulgaris* herb contains essential oils (0.07–0.20%), which contains: limonene, terpinolene, fenchone, aromadendrene, thayyl alcohol, thujone (alpha and betta), alpha-pinene, beta-pinene, camphor, and others [49]; triterpenoids: alpha-amyrin, fernenol; steroids: beta-sitosterol, stigmasterol; amino acids: arginine, histidine, asparagine, proline, lysine, alanine, valine, glycine, isoleucine, aspartic acid, methionine; phenolcarboxylic acids (not less than 0.5% in terms of chlorogenic acid); coumarins (1.2%): scopoletin, umbelliferone, imperatorin, esculetin, xanthotoxol, coumarin [48]; flavonoids (not less than 0.5% in terms of rutin): quercetin, kaempferol, isorhamnetin, apigenin; carbohydrates: polysaccharides, inulin, starch [49]; and artemisinin, a substance with antioxidant properties, was also discovered in the herb [50].

One study [51] investigated the effect of various factors on the yield, antioxidant activity (AA), and total phenol content (TPC) of plant extracts (guava leaves). The effect of leaf sample pretreatment before extraction, extraction method, and leaf age were studied. The results showed that sonication is the most suitable method for plant extraction as it produces an extract with a significantly higher AA. Blanching followed by cooling with ice water was proposed for the leaf pretreatment process. The study of leaf maturity showed that young leaves show the greatest activity. Hot water was the best solvent for extracting the active ingredients. An aqueous extract of young leaves pre-treated by blanching and cooling showed the highest AA values. These values are 1.88-times higher than those of the synthetic antioxidant butylated hydroxytoluene. It was concluded that the extraction of plant extract bioactive components and their antioxidant capacity is influenced by pre-treatment and drying processes, extraction method, and leaf maturity [51].

## 3. Anti-Neurodegenerative Properties of Medicinal Plants and Their Complexes

Millions of patients in the world suffer from chronic neurodegenerative diseases (Parkinson’s and Alzheimer’s diseases, Huntington’s chorea, hyperprolactinemia, etc.). The key link in the pathogenesis of neurodegenerative diseases is the degeneration of specific neurons, which over time leads to dysfunction in the regulation of which they are involved—cognitive functions in Alzheimer’s disease, motor behavior in Parkinson’s disease, etc. [52].

Alzheimer’s disease (AD) has a special place among neurodegenerative diseases (ND) in terms of its negative significance for society [52]. According to the World Health Organization experts, AD is the most common cause of dementia in the elderly. The global prevalence of dementia in the world will practically double every 20 years to 65.7 million in 2030 and 115.4 million in 2050. A particularly sharp increase in patients will occur in middle- and low-income countries. The prevalence of the disease increases as the age category increases. In people older than 65 years, the number of patients doubles every five years. The available statistical data give grounds to consider AD, along with cardiovascular and oncological diseases, as one of the most serious medical problems in developed countries [53]. The risk of developing Alzheimer’s disease is significantly higher in women than in men, mainly due to the higher life expectancy of women compared to men [53].

The etiology of Alzheimer’s disease is still an open question. Currently, factors and diseases have been identified that increase the risk of AD developing. Risk factors are advanced age, obesity, insulin resistance, vascular factors, dyslipidemia, hypertension, CNS traumatic injury, and depression. The anatomical pathology of AD at the microscopic level includes neurofibrillary tangles (NFT), senile plaques (SP), and cerebrocortical atrophy, which mainly develops in the association regions and medial areas of the temporal lobe. Alzheimer’s disease is accompanied by proteinopathy—the accumulation in the brain tissues of abnormally folded proteins—of amyloid-beta and tau protein. Plaques are formed from small peptides 39–43 amino acids long called amyloid-beta (A-beta, Aβ). Amyloid beta is a fragment of a larger precursor protein, APP. This transmembrane protein plays an important role in neuron growth, survival, and recovery from damage. In Alzheimer’s disease, for unknown reasons, APP undergoes proteolysis—it is divided into peptides under the influence of enzymes. Aβ strains formed by one of the peptides stick together in the intercellular space into dense formations known as senile plaques [54]. In Alzheimer’s disease, changes in the structure of the tau protein lead to the disintegration of microtubules in brain cells. More specifically, Alzheimer’s disease is also referred to as tauopathies, diseases associated with abnormal aggregation of the tau protein. Each neuron contains a cytoskeleton made up of microtubules that carry nutrients and other molecules from the center to the periphery of the cell, to the end of the axon, and back. Microtubules are made up of the tau protein, which stabilizes them along with several other proteins once they are phosphorylated. In Alzheimer’s disease, tau protein is over-phosphorylated.

Herbal preparations can play an important role in the prevention and treatment of neurodegenerative diseases, including Alzheimer’s and Parkinson’s diseases. This review summarizes research by scientists worldwide on these diseases. Many studies by international and Russian scientists have relied on preliminary and clinical studies [55,56,57]. Alzheimer’s and Parkinson’s diseases are among the major neurodegenerative disorders (NDDs) that impose a significant socioeconomic burden. People have been searching for a cure for NDDs using natural herbs for centuries. It is reported that many medicinal plants and their secondary metabolites are able to alleviate the symptoms of NDDs [58,59]. The major identified mechanisms by which phytochemicals exert their neuroprotective effects and potential neurological health support during aging include antioxidant, anti-inflammatory, antithrombotic, antiapoptotic, acetylcholinesterase and monoamine oxidase inhibition, and neurotrophic activity [60,61]. Studies [62,63] reviewed clinical trials and provided statistical data on the mechanisms of action of some major herbal products with potential in the treatment of NDD, according to their molecular targets, as well as their regional sources. A number of studies have demonstrated the beneficial properties of plant extracts or their bioactive compounds against NDDs [64,65]. Plant products potentially offer new treatment options for patients with NDD that are a cheaper and culturally acceptable alternative to traditional therapies for millions of people around the world with age-related NDDs [66,67].

The mechanisms of this effect are not always known. Perhaps antioxidant, adaptogenic mechanisms play a role here. Due to the content of carotenoids, some medicinal plants and their complexes prevent the occurrence of Alzheimer’s disease. This fact was evaluated in a number of studies [68,69,70,71,72]. Alzheimer’s disease is the most devastating neurodegenerative disease affecting the aging population worldwide. Endogenous and exogenous factors are involved in the triggering of this complex and multifactorial disease, the hallmark of which is amyloid-β (Aβ), formed as a result of the breakdown of the amyloid precursor protein under the action of β- and γ-secretase. Although there is no cure for Alzheimer’s disease at this time, many neuroprotective natural products, such as polyphenol and carotenoid compounds, have shown promising preventive activity as well as helping to slow the disease’s progression [68]. Studies [68,70] have focused on the chemistry as well as the structure of carotenoid compounds and their neuroprotective activity against Aβ aggregation using molecular docking assays. Besides the most common anti-amyloidogenic carotenoid, lutein, cryptocapsin, astaxanthin, fucoxanthin, and the apocarotenoid bixin have all been studied [70]. Structure-based computer analysis of drug design and molecular docking simulations have revealed important interactions between carotenoids and Aβ through hydrogen bonding and van der Waals interactions and have shown that carotenoids are potent anti-amyloidogenic molecules with a potential role in preventing Alzheimer’s disease, especially since most of them can cross the blood–brain barrier and are considered nutraceuticals [71]. As a result of these findings, we now have a better understanding of how carotenoids inhibit Aβ aggregation. The potential role of carotenoids as new therapeutic molecules in the treatment of Alzheimer’s disease and other neurodegenerative diseases has been discussed [72].

Nobiletin and tangeretin, flavonoids isolated from the peel and other parts of citrus fruits, have a neuroprotective effect in experiments in vitro and in vivo and are promising in the prevention and treatment of Alzheimer’s and Parkinson’s diseases [73]. Citrus naringenin prevents dopamine synthesis disorders in the brain which prevents the development of Parkinson’s disease. Sedative, muscle relaxant, anti-hallucinogenic, neuroprotective, memory-enhancing, and anti-Parkinsonian properties of medicinal plants have been noted.

Polyphenols, due to the antioxidant, anti-inflammatory properties of medicinal plants and their extracts, have a neuroprotective effect in epilepsy and other neurodegenerative diseases; resveratrol and flavonoids prevent the occurrence and development of neurodegenerative diseases and have a neuroprotective effect. Due to its antioxidant, anti-inflammatory properties, methanol extract of ginger root can serve as an additional tool in the treatment and prevention of Alzheimer’s disease [74]. Experimental studies have shown that taking cinnamon powder (*Cinnamonum cassia*, *Cinnamonum verum*) prevents T-cell dysfunction, thereby providing a therapeutic and prophylactic effect in autoimmune diseases, including multiple sclerosis [75]. Lemon oil reduces lipid peroxidation in the hippocampus, thereby preventing the development of neurodegenerative diseases [76]. Experimental studies have shown the presence of neuroprotective properties in *Terminalia chebula* Retz. seed extracts [77]. Chebulic acid has pronounced neuroprotective properties [78]. Ellagic acid has the same properties [78]. *Silibum marianum* Gaerth silubin has immunosuppressive properties, and this makes it possible to use it in multiple sclerosis [79]. Silubin prevents memory impairment and destruction of nerve cells caused by oxidation, which opens up prospects for its use in the treatment of Alzheimer’s disease [80]. Ferulic acid (*Ferula assa foetida* L.), due to its antioxidant properties, has a therapeutic effect in neurodegenerative diseases such as Alzheimer’s and Parkinson’s diseases [81].

Experimental studies have shown that alcoholic extracts of date fruits (*Phoenix dactylifera* L.) have a neuroprotective effect in ischemic damage to the nervous tissue [82]. Experimental studies have shown that the date diet, due to its antioxidant properties, can serve as a prophylactic for Alzheimer’s disease [83]. As we have already noted, acetyl and butyrylcholinesterase enzymes play an important role in the pathogenesis of the development of complications from the nervous system in Alzheimer’s disease. In the plant world, biologically active substances that inhibit these enzymes are common. The hot infusion of orange (*Citrus sinensis* (L.) Osbeck.) peel has been found to inhibit MAO and butyrylcholinesterase, which opens up great prospects for its use in the treatment of neurodegenerative diseases [84]. It has also been determined that aqueous extracts of oranges inhibit acetylcholinesterase, and they can serve as a therapeutic agent in the treatment of Alzheimer’s disease [85]. Anticholinesterase activity has been shown for a number of natural compounds, due to which they can be used for the prevention and treatment of Alzheimer’s disease: essential oil of leaves and flowers of *Polygonum hydropiper* L., phenolics and flavonoids in *Inula britannica* L. [86,87], rosmarinic acid from extracts of *Hypericum perforatum* L. [88], phenolics from extracts of *Terminalia chebula* Retz [89], alkaloids of *Fumaria vailantii* Loisl. [90], extracts *Coriandrum sativum* L. [91], a mixture of chokeberry and lemon juices [92], ursolic acid and oil of *Origanum majorana* L. [93], extracts of *Myristica fragrans* Houtt. [94], alcohol *Foeniculum vulgare* Mill. fruit extracts and its oil [95], extracts of *Thymus serpyllum* L. [96], extracts of *Rumex confertus* Willd. leaves [97], mulberry root bark extracts [98], *Pleurotus ostreatus* (Fr.) [99], castor bean leaf extracts [100].

The stems and fruits of black pepper (*Piper nigrum* L.) have shown the ability to inhibit acetylcholinesterase, and butyrylcholinesterase. Cucumber fruit extracts have anticholinesterase and antimonoamine oxidase activity, which opens up prospects for their use in the treatment of neurodegenerative diseases [101]. Substances that prevent the formation of amyloid fibrils have been identified among the biologically active substances of plants. Grape seed gallic acid prevents the formation and accumulation of amyloid fibrils, which play the main pathogenetic role in Alzheimer’s and Parkinson’s diseases [102]. Experimental studies have revealed the properties of alcoholic extracts of buckwheat (*Fagopyrum esculentum* Moench.) to inhibit the production of β-amyloid and prevent memory impairment [103]. In the pathogenesis of Alzheimer’s disease, deficiency of P-glycoprotein with adenosine transferase protein (ABCB1), which is involved in the transport of β-amyloid from the brain tissue into the blood, plays an important role. Experimental studies have shown that *Hypericum perforatum* L. extracts increase the rate of transport of β-amyloid into the blood, thereby providing a preventive and therapeutic effect in Alzheimer’s disease [104]. Similar properties have been found in curcumin.

Quinolinic acid is formed due to the degradation of tryptophan. The accumulation of this substance stimulates the processes of neuroinflammation and demyelination, and the development of degenerative diseases such as multiple sclerosis. Experimental studies have shown that alcohol *Terminalia chebula* Retz. extracts inhibit the accumulation of this substance and the development of oxidative stress in the nervous tissue under the influence of quinolinic acid [105]. Calamus (*Acorus calamus* L.) root preparations [106] and asparagus (*Asparagus officinalis* L.) extracts [107] reduce damage to the nervous tissue by β-amyloid, thereby preventing the development of Alzheimer’s disease. Experimental studies have shown that curcumin (*Curcuma longa* L.) prevents degradation caused by toxic factors of nigral dopaminergic neurons and prevents the development of Parkinson’s disease [108]. Methanol extracts of black pepper (*Piper nigrum* L.) reduce oxidative stress in the hippocampus under the influence of β-amyloid [109]. In addition, pepper fruits improve memory by stimulating the trophism of the nervous tissue, especially in the hippocampus. *Acorus calamus* L. β-azarone is considered a potentially effective agent in the treatment of neurodegenerative diseases, including Alzheimer’s disease [110]. Ginseng preparations have been shown to be effective in the treatment of Parkinson’s disease due to their neuroprotective properties of ginseng [111].

Experimental studies have shown that taking extracts of *Hypericum perforatum* L. has a therapeutic effect in Parkinson’s disease [112]. Plant extracts are also promising as a therapeutic agent for multiple sclerosis [113]. A similar effect was shown for cinnamon (*Cinnamomum Blume* L.) extract [114]. A number of medicinal plants and their metabolites have a therapeutic effect in Alzheimer’s disease: nobiletin and narirutin, flavanoids from lemon fruits [115,116]; tangeretin has therapeutic potential in inflammatory and degenerative processes in the nervous tissue accompanied by microglial activation [117]; seeds and roots of *Peganum harmala* L. [118], *Crocus sativus* L. stigmas [119], *Trigonella foenum-graecum* L. seeds [120,121]. Experimental studies have shown that fenugreek seed extracts have a therapeutic effect on motor disorders in animal models of Parkinson’s disease. Randomized, placebo-controlled clinical trials have shown that IBHB (fenugreek seed extract) is a safe, effective adjuvant treatment for patients with L-DOPA-dependent Parkinson’s disease.

*Filipendula ulmaria* L. *Maxim*, due to the presence of flavonoids such as kaempferol, luteolin, and apigenin, has a therapeutic effect in neurodegenerative diseases such as Alzheimer’s disease, Parkinson’s disease, epilepsy, multiple sclerosis, and stroke [122]. Animal experiments have shown that meadowsweet has a therapeutic effect and prevents the processes of demyelinization in encephalomyelitis [123]. Glabridin, extracted from mistletoe (*Víscum álbum* L.), protects against the deterioration of cognitive processes and memory caused by exposure to chemical agents. This opens up prospects for the use of glabridin in the treatment of Alzheimer’s disease. Neuroprotective properties of rosehip extracts (*Rosa cinnamonea* L.) have been noted. Experimental studies have shown that a herbal preparation of rose hips, tansy herb, and nettle prevents memory impairment in Alzheimer’s disease [124]. *Valeriana officinalis* L. extract improves cognitive functions caused by exposure to amyloid β in models of Alzheimer’s disease [125]. The anti-neurodegenerative effects of various plant components are summarized in Table 1. The table demonstrates a fairly wide range of plants of various species that exhibit significant antioxidant activity. Many plant species considered include neurodegenerative and antioxidant components.

Table 1 demonstrates only the main components of the presented plants that affect the antioxidant activity. It should be noted that these plants also contain other components (for example, ellagitannins in *Filipendula ulmaria* or procyanidins in *Vitis vinifera* and wine), which have a beneficial effect on the oxidation of free radicals, antioxidant activity, and the aging process of the human body [122,123].

Essential oil is one of the key components of *Citrus limon*. Lemon essential oil is extracted by cold pressing the fruit’s peel. The oil is fractionated by vacuum distillation, and essential oils with varying concentrations of the main components (limonene, other monoterpene hydrocarbons, citral, geranyl acetate, and sesquiterpenes) are produced. The three primary components responsible for the aroma of lemon are limonene (1-methyl-4-(1-methylethenyl)cyclohexene) and citral (a combination of two geometric isomers of 3,7-dimethyl-2,6-octadienal, neral and geranial). It was demonstrated that citral is unstable; it is easily oxidized in the presence of light or oxygen [133]. In addition to these components, lemon essential oil contains α-pinene, sabinene, β-pinene, β-myrcene, p-cymene, α-terpinene, limonene, γ-terpinene, linalool, neral, geranial, neryl acetate, and geranyl acetate. Lemon essential oils are powerful antioxidants. The inhibitory effect of essential oil components on model aldehyde oxidation is based on competitive reactions between these components and the aldehyde with an oxidizing agent, in this case air oxygen. The components of essential oils oxidize and transform, resulting in a change in essential oil composition, the emergence of new products, and the consumption of the main components. On the other hand, comparing the rate of oxidation (decrease in concentration) of oil components allows for evaluation of their antioxidant activity.

It was found that the antioxidant activity depends on the composition of the systems and on the concentration of essential oils. Citral and limonene had the lowest antioxidant activity; a mixture of these compounds had a higher activity. The antioxidant properties of essential oils increased as their concentration or the content of monocyclic terpene hydrocarbons in the model systems increased, especially α- and γ-terpinenes. Differences in the oxidation resistance of the main components of lemon essential oils were determined, and synergistic effects in the antioxidant activity and stability of essential oil components were found [133].

*Rauwolfia serpentina* Benth., a tropical plant from the *Apocynaceae* family, is a source of many indole alkaloids that are widely used in medical practice as antihypertensive, antiarrhythmic, and sedative drugs. Ajmaline is a key component among the alkaloids of this plant [134]. Ajmalin is an antiarrhythmic medication that is effective in the treatment of the cardiovascular system. Due to the endemic and endangered status of *Rauwolfia serpentina*, as well as its slow growth rate and relatively low content of ajmaline alkaloids, these compounds are currently obtained from in vitro cultivated tissues of this plant. Indole alkaloids *Rauwolfia serpentina* are also represented by reserpine, serpentine, rescinnamine, or yohimbine. *Rauwolfia* alkaloids have various pharmacological properties. They mainly affect the central nervous system [134].

One of the modern directions of pharmaceutical research is the search for new types of plant materials to expand the range of fatty oils for medical use in aging. A promising source of fatty oils are the seeds and fruits of oleaginous plants. In particular, pomegranate seeds are a source of fatty oil of an atypical chemical composition, which has been found to contain the following fatty acids: palmitic (C16:0), linoleic (C18:2, cis), oleic (C18:1, cis), linolelaidic (C18:2, trans), stearic (C18:0), punicic (C18:3, 9cis, 11trans, 13cis), α-eleostearic (C18:3, 9cis, 11trans, 13trans), catalpic (C18:3, 9trans, 11trans, 13cis), β-eleostearic (C18:3, 9trans, 11trans, 13trans), eicosene (C20:1, cis), arachidonic (C20:0), lignoceric (C24:0), and conjugated linolenic acids [135]. Thus, pomegranate oil consists of triglycerides containing unsaturated fatty acids, mainly punicic acid, which has a pronounced positive biological effect on the body [135].

The study of *Terminalia chebula* revealed its antioxidant, antibacterial, antitumor, neuroprotective, anti-inflammatory, antidiabetic, hepatoprotective, antimutagenic, antiproliferative, radioprotective, cardioprotective, antiarthritic, anticaries, and wound healing properties [136]. *Terminalia chebula* fruits contain 1,2,3-tri-O-galloyl-6-O-cinnamoyl-β-d-glucose, 1,2,3,6-tetra-O-galloyl-6-O-cinnamoyl-β-d-glucose, 4-O-(2″,4″-di-O-galloyl-α-l-rhamnosyl)ellagic acid, 1’-O-methyl neochebulin, dimethyl neochebulinate, 6’-O-methyl neochebulinate, dimethyl neochebulinate, dimethyl 4’-epineochebulinate, methyl chebulate, derivatives of polyhydroxytriterpenoids-23-O-neochebuloylarjungenin 28-O-β-d-glycopyranosyl ester, 23-O-4’-epi-neochebuloylarjungenin, and 23-O-galloylpinfaenoic acid 28-O-β-d-glucopyranosyl ester. The hydrolysable tannins chebumeinin and chebumeinin B were also found [137,138,139]. The bark of the plant contains triterpenoids-termichebuloside, dimeric triterpenoid saponin, termichebulolide, oleanolic acid-type lactone [140]. The plant contains Cl, Co, Cr, Fe, K, Mn, Na, Se and Zn salts [141]. Plant seed extracts have been demonstrated to exhibit neuroprotective effects in experimental studies. Chebulic acid has pronounced neuroprotective properties [142]. Ellagic acid from Chebulic myrobalans has the same properties [143]. Plant extracts are promising for the treatment of Alzheimer’s disease because of their antioxidant and anti-inflammatory characteristics [138]. Experiments have demonstrated that plant extracts suppress the enzyme acetylcholinesterase, which is involved in the progression of Alzheimer’s disease [144,145]. Quinolinic acid is formed due to the degradation of tryptophan. The accumulation of this substance stimulates the processes of neuroinflammation and demyelination, and the development of degenerative diseases such as multiple sclerosis. Experimental studies have shown that alcoholic extracts of Chebulic myrobalans inhibit the accumulation of this substance and the development of oxidative stress in the nervous tissue under the influence of quinolinic acid [146].

The healing properties of *Sílybum mariánum* have been known since ancient times. Both the plant and the extract obtained from it are utilized in folk medicine. *Silybum marianum* has a number of protective properties against liver diseases and neurogenerative diseases [147]. It is a natural antioxidant. *Sílybum mariánum* seeds contain flavonoids from which a pharmacological agent silymarin is produced. Silymarin contains three main components: flavonoids silybin, silychristin, and silydianin. Silymarin has an antioxidant effect in Alzheimer’s disease. The seeds are rich in fatty oils (20–30%), proteins (25–30%), also contain tocopherol (0.038%) and sterols (0.63%) including cholesterol, campesterol, stigmasterol, sitosterol, and others. Fatty oils are an indispensable nutritional factor that provides a person with essential fatty acids (linoleic, linolenic, and arachidic), which are not synthesized in the human body. They are also used in the production of medicines. The identified new fatty oils containing a high amount of essential fatty acids (C18:2, 18:5) belonging to the class of ω-2, ω-3 acids are of great interest [147].

*Inula britannica* contains flavonoids, essential oils, carotene, sesquiterpene lactones, including britanin, tannins, and other substances. The seeds contain traces of alkaloids, and the roots contain the bitter substance insulin and other compounds, which has led to their widespread use in medicine (official, folk, veterinary) and food industry [148]. Components typical of the *Asteraceae* family as a whole were detected in the glandular trichomes secretion of *I. britannica*—essential oils including sesquiterpene lactones and phenolic compounds (flavonoids). The fact that the content of trichomes stain with methylene blue, which belongs to the group of basophilic dyes, indicates the predominance of oxidized components in the composition of the essential oil (sesquiterpene lactones, flavonoids, phenolic acids). The glandular trichomes of *I. britannica* synthesize essential oils (at the initial stages of formation) and oleoresins (formed). The secret of the glandular trichomes of I. britannica is formed by a polysaccharide mucus, which includes neutral sugars (the secret is not stained with methylene blue); the content of numerous non-glandular trichomes is filled with essential oil, which is released when the terminal cell is broken off [149].

*Fumaria officinalis* contains about 1% of a mixture of isoquinoline (styloptin, protopin, cryptopin, synactin, bicuculin, adlumin, etc.) and spirobenzylisoquinoline (fumarophycin, parfumin, fumaritrin, etc.) alkaloids [150,151]. Alkaloids adlumicein methyl ester, parfumine, and N-methylhydrastine methyl ester were identified in *Fumaria officinalis* [150]. In *Fumaria officinalis*, isoquinoline alkaloids protopine, cryptopine, fumaranine, fumarostrezhdin, parfumidin, synactin, etc. were identified. These substances have a neurodegenerative effect in Alzheimer’s disease and aging [151]. Glycosides, vitamins C, K, organic acids, sugars, resins, traces of essential oil were also found. Spirobenzylisoquinoline alkaloid fumariline was determined in seeds [150,152].

Vitis vinifera is a promising source of biologically active substances. Along with fruits, which are valuable medicinal plant raw materials, leaves are often used for the manufacture of a number of medicines and biologically active food supplements, which have antioxidant, cardiovascular, anti-sclerotic, capillary-strengthening, and anti-inflammatory effects [153]. Twenty compounds belonging to different chemical groups were identified in the lipophilic fraction: (a) high molecular weight fatty acids and their derivatives (myristic, palmitic, linolenic, palmitoleic acids); (b) compounds of terpene nature, which are an integral part of the essential oil fraction (d-limonene, geranyl, phenylacetate, squalene, etc.); (c) diterpene alcohol phytol; (d) fat-soluble vitamins α- and γ-tocopherols; (e) a pyrazole derivative; (f) higher aliphatic hydrocarbons and other organic substances [153].

*Curcuma longa* L. has a wide range of biological effects, including anti-inflammatory and antioxidant properties. Its specific effect on various organs and tissues has been revealed, i.e., on the skin, gastrointestinal tract, liver, and respiratory system. As part of the components of turmeric, carbohydrates (4.7–8.2%), essential oils (2.44%), fatty acids (1.7–3.3%), and curcuminoids (curcumin, demethoxycurcumin, and bisdemethoxycurcumin) have been isolated, the content of which is approximately 2%, although they can reach 2.5–5.0% of dry weight, as well as other polypeptides such as turmerin (0.1% dry extract) [154]. One of the main active components of *Curcuma longa* L. is curcumin, a polyphenol, the main representative of the curcuminoids group. The antitumor, antioxidant, and anti-inflammatory activity of curcumin has been confirmed. Curcumol, a component of turmeric essential oil, has anti-epileptic properties. Anti-epileptic properties were determined in the bisabolene terpenoids of turmeric. In experimental animals, curcumin reduced movement disorders and stiffness caused by haloperidol. Experimental studies demonstrated that curcumin prevents the degradation caused by toxic factors of nigral dopaminergic neurons and prevents the development of Parkinson’s disease. Curcumin has a positive therapeutic effect in all neurodegenerative diseases [154].

Biologically active substances of *Acorus calamus* are represented by essential oil, polysaccharide complex, phenolic compounds [155]. The composition of the essential oil includes: d-alpha-pinene, d-camphene, d-camphor, borneol, eugenol, methyleugenone, azaron, beta-azaron, calamen, sesquiterpene ketone akorone, caryophyllene, proazulene, and other terpenoids. *Acorus calamus* is used for the treatment and prevention of cardiovascular diseases, neurodegenerative disorders, epilepsy, Parkinson’s, and Alzheimer’s diseases [155].

The medicinal plant *Filipendula ulmaria* is a source of highly effective preparations of various actions: anti-inflammatory, immunostimulating, antitumor, antioxidant, adaptogenic, and nootropic [156]. *Filipendula ulmaria* contains isobutylamine, isoamylamine, higher fatty acids (stearic and lenolenic), hexanal, 6,10,14-trimethyl-2 peptadecanone, 2 nonadecanone, 14-methyl pentadecanoic acid ester, dodecanoic, tetradecanoic, pentadecanoic and hepatodecanoic acids, 1-nonadecene, hexadecanoic acid ester, 1-octadecanol, 9,12 octadecadienoic acid [156].

The chemical composition of *Viscum* species, in particular *Viscum album*, has not been sufficiently studied, but it is known that their medicinal effectiveness is due to the content of a number of chemically complex and diverse active substances. These include polypeptides, carbohydrates, amines, organic acids (lactic, isovaleric, caproic, etc.), rubber steroids, cardenolides and triterpene glycosides (viscumneoside V, naringenin, rhamnocitrin, etc.), phenols, higher fatty acids, saponins, and many others [28]. For *Viscum album*, 41 components of the extract have been presented, which include 11 flavonoids, 2 hormones, 14 benzenoids, 1 inositol, 2 pyrimidines, 4 triterpenoids, 5 steroids, viscoline, and a new flavonone-(2S)-7,4’-dihydroxy-5, 3’-di-methoxyflavanone [157]. White mistletoe is used as an analgesic, astringent and enveloping agent, for the treatment of hypertension, gastrointestinal, uterine and hemorrhoidal bleeding, metabolic disorders, early menopause in women, dehelmintization, as well as an anti-inflammatory, anticancer, and antioxidant agent [157].

## 4. Anti-Inflammatory Properties of Medicinal Plants and Their Complexes

It is estimated that more than 150,000 plant species have been studied, many of which contain valuable therapeutic agents, and the use of new compounds from plants for pharmaceutical purposes has been gradually increasing in recent years [158]. Since ancient times, plants have played an important role in protecting human health. When adapting against pathogen attack and environmental stress, plants produce several substances that exhibit biological activity. These organic molecules are secondary metabolites and also exhibit biological activity. Among the various functions, the anti-inflammatory effect of plants is distinguished. It is known that inflammation is an evolutionarily conserved defense process and a critical survival mechanism [159]. It consists of complex successive changes in tissue aimed at eliminating the original cause of damage to the cell, which could be caused by infectious agents or substances released during their metabolism (microorganisms and toxins), as well as physical factors (radiation, burns, and injuries), or chemicals (caustic substances). Signs of inflammation are local redness, swelling, pain, burning, and loss of function [160].

In general, this complex biological response leads to the restoration of homeostasis. However, in cases of sustained release of inflammatory mediators and activation of insecure signaling pathways, the inflammatory process persists, and a chronic pro-inflammatory state may occur [161]. Chronic inflammation may be associated with diseases such as obesity, diabetes, cancer, and cardiovascular disease. Medicinal plants play an important role in the development of new potent anti-inflammatory drugs [162]. Ethnobotanical research made it possible to combine a variety of plants with biological activity by methods of observation, description, and experimental studies, which greatly contributed to the discovery of natural plant products of biological action. The use of plant-based medicinal natural compounds for the treatment of many diseases has become a trend in modern clinical research. Polyphenolic compounds have attracted significant attention due to their modulating effect on inflammasomes [163]. These multiprotein complexes are associated with the onset and progression of metabolic disorders and chronic diseases caused by inflammation [163].

Over the past decades, hundreds of research and review articles have been published on the anti-inflammatory activity of plants [164]. It is important to note that the extraction of plant raw materials is an important step that makes it possible to obtain a preparation with a specialized action [164]. When a set of natural compounds is used, there is a high possibility of synergy between the active ingredients, which can be lost when each of these ingredients is isolated. This synergism has been found in several medical tests, including anti-inflammatory activity. On the other hand, a mixture of different compounds can also lead to inhibitory effects, namely that one component can reduce the biological activity of another. Medicinal plants are used instead of non-steroidal anti-inflammatory drugs, given that the use of these drugs is associated with undesirable effects on the gastrointestinal tract and kidneys. The biggest disadvantage of strong synthetic drugs is their toxicity and recurrence of symptoms after withdrawal. Thus, screening and development of herbal preparations with anti-inflammatory action are currently needed, and much effort is being made to find anti-inflammatory preparations from medicinal plants [165]. Substances of plant origin belonging to various chemical classes have demonstrated proven anti-inflammatory activity [156]. Among them are alkaloids, terpenes [157], phenolic compounds, tannins, lignans, coumarins, saponins, and especially flavonoids [152].

The *Álnus incána* flavonoids—rutin, quercetin, and hesperidin—have been found to have an anti-inflammatory effect [153]. Studies involving the *Potentilla argentea* glycosides (kaempferol, quercetin, aromatendrenene) showed anti-inflammatory activity due to the suppression of NO levels in microglial cells [154]. *Agrimónia eupatória* terpenes, which exhibit pharmacological properties such as anti-inflammatory and antinociceptive abilities, inhibit platelet aggregation and interfere at the intracellular level with the transduction mechanism [155]. These compounds also contribute to a significant reduction in edema and exhibit effects comparable to those of hydrocortisone. Some pathologies, such as inflammation, can be exacerbated by the formation of free radicals that cause tissue damage by promoting oxidation [156]. Oxidative stress is known to play an important role in endothelial dysfunction; lung disease, gastrointestinal dysfunction, atherosclerosis, and inflammatory symptoms are implicated in all these disorders [157]. Excessive pro-inflammatory cytokines and mitochondrial dysfunction cause oxidative stress, characterized by an imbalance between the effectiveness of antioxidant protection and the rate of formation of reactive oxygen species, causing an overload of oxidants [158].

Antioxidant compounds can reduce oxidative stress, minimizing the incidence of pathologies. The search for new antioxidant agents from plant sources used against inflammation and infection may lead to the discovery of natural molecules with high anti-inflammatory potential in vitro and in vivo. These substances justify the popular use of these plant species with anti-inflammatory properties [166]. Thus, arachidonic acid metabolites play a vital role in inflammation. In the inflammatory process, arachidonic acid is released from membrane phospholipids by the enzyme phospholipase and metabolized by cyclooxygenases, lipoxygenases, and cytochromes into prostaglandins/thromboxane, leukotrienes, and epoxy/hydroxy metabolites, such as epoxyeicosatrienoic acid. Cyclooxygenase (COX), the enzyme responsible for the formation of prostaglandins from arachidonic acid, is released from cell membrane phospholipids by phospholipase [167]. COX is necessary to maintain the normal physiological state of many tissues, including protecting the gastrointestinal mucosa, controlling renal blood flow, homeostasis, autoimmune and anti-inflammatory responses, and controlling the functions of the pulmonary, nervous, and cardiovascular systems, and the reproductive functions of the human body [168]. COX expression is significantly increased during inflammation, or mitogenic stimulation [167] induced by inflammation by cytokines and endotoxins, and causes a decrease in the number of prostaglandins that contribute to the development of edema, hot flashes, fever, and hyperalgesia [168]. Therefore, activation of these enzymes stimulates intracellular signals that alter the expression of pro-inflammatory cytokines such as interleukin. COX inhibition is regarded as an important target for potential drugs for the treatment of inflammatory processes in the aging human body [169]. Inhibition of COX by plant BAS is responsible for the imbalance of arachidonic acid metabolites; plant BAS increase the production of lipoxygenase products, leukotrienes, which have pro-inflammatory properties [164]. Glycation or non-enzymatic glycosylation is a reaction between reducing carbohydrates (glucose, fructose, etc.) and free amino groups of proteins, lipids, and nucleic acids of a living organism, proceeding without the participation of enzymes. Glycation is a special case of the Maillard reaction. Non-enzymatic glycosylation of proteins is a key mechanism of tissue damage in diabetes mellitus [165].

## 5. Antiglycating Properties of Medicinal Plants and Their Complexes

The glycation process, which is enhanced by hyperglycemia, underlies the pathogenesis of micro and macrovascular complications of diabetes mellitus (DM), which is common in the elderly [166]. Glycosylation end products (GEP) affect type IV collagen, myelin, tubulin, plasminogen activator-1, and fibrinogen. The receptor-dependent effects of GEP are mediated by their interaction with specific receptors, which leads to the activation of the nuclear factor NF-κB, which moves to the nucleus and leads to an increase in the transcription of intercellular adhesion molecules-1, E-selectin, endothelin-1, vascular endothelial growth factor, and pro-inflammatory cytokines [167]. The first and most studied substance that inhibits protein glycation is aminoguanidine, which prevents the formation of GEP [168]. However, clinical trials of this drug were stopped due to the lack of efficacy and the presence of side effects (gastrointestinal symptoms, lupus-like, flu-like syndromes, vasculitis, anemia). Antiglycation activity was found in pyridoxamine, hydrazine derivatives of thiazolidine and carboxymidamide, structurally similar to aminoguanidine, and derivatives of phenoxyisobutyric acid [169]. All of the above determines the relevance of the search for plant substances that prevent the formation of GEP in order to create drugs for the pathogenetic prevention of DM complications.

Diabetes mellitus is a metabolic disorder characterized by hyperglycemia. The prevalence of diabetes and its associated complications has increased dramatically over the past few decades, leading to increased morbidity and premature mortality, and remains a major risk factor for cardiovascular disease worldwide [170]. Chronic hyperglycemia is the main factor causing vascular and internal organ damage in diabetes [171]. An uncontrolled excess of glucose in the blood reacts with the free amino acids of proteins to form a labile Schiff base and is stabilized in a compound known as GEP. Protein glycation causes several structural modifications and alters the function of many proteins, especially albumin. This process affects the affinity of the albumin-binding activity of drugs, hormones, fatty acids, and other substances [172]. In addition, albumin-derived GEPs have been shown to trigger the generation of intracellular reactive oxygen species, which leads to inhibition of glucose uptake and oxidative changes in intracellular proteins [173,174]. Albumin has the ability to scavenge free radicals depending on its structure, and this protective function is lost in uncontrolled diabetes [175]. Numerous GEP inhibitors, including pharmacological and natural compounds, have been investigated for their ability to prevent the complications of diabetes, but medicinal plants are considered safer than others, and many of them have the ability to reduce the harmful effects of hyperglycemia [176]. Widespread in Europe, *Solidágo virgáurea* L. and *Lamium album* L. have played a traditional role in folk medicine for centuries in the treatment of skin diseases, rheumatism, hypertension, and various infections. These plants are rich in phenolic flavonoids and phenolic acids. Many compounds of these plants have antioxidant properties that act through enzymatic and non-enzymatic pathways [177]. Recent studies have demonstrated the antibacterial and antioxidant effects of *Solidágo virgáurea* L. and *Lamium album*, as well as their hypoglycemic effects in diabetes [178]. It was found that the anti-glycation properties of the extracts of *Solidágo virgáurea* L. and *Lamium album* were strongly correlated with their antioxidant capacity (trapping DPPH-radicals). Like antioxidant capacity, anti-glycation activity correlated strongly with phenol and flavonoid content.

Phenols and flavonoids are classified as antioxidants and have been reported to have protective effects in diabetes. Some studies show that a higher content of phenol and flavonoids has a greater effect on protection against hyperglycemia [179]. Albumin glycation, including the formation of fructosamine, carbonyl groups, and amyloid β-structures, is significantly attenuated in the presence of plant extracts. The initial step in the formation of GEP, also known as the Maillard reaction, begins with the nucleophilic addition of the free amino groups of proteins to the carbonyl group of reducing sugars to reversibly form a Schiff base product, which in turn is converted to a stable fructosamine residue (ketoamine) by Amadori rearrangement (Figure 2). The Schiff base and fructosamines are called early glycation products. These adducts can undergo subsequent oxidation, rearrangement, dehydration, and cyclization to form stable agents called GEP [172]. It has been demonstrated that GEP inhibitors can prevent the formation of reactive dicarbonyls and oxygen species [169]. It has been suggested that antioxidants such as phenols and flavonoids inhibit the formation of GEP and such properties have been attributed to the structure of these compounds. In fact, adjacent OH groups have been found to be responsible for their antioxidant and antiglycation activity [173]. The relationship between antioxidant and antiglycation activity was evident in that the maximum inhibition of glycation was noted for medicinal plants, which showed the highest antioxidant properties. *Solidágo virgáurea* and *Lamium album* showed a low ability to prevent the formation of amyloid β-structures. The research results show that the extracts and complexes of these plants are able to scavenge free radicals and prevent albumin glycation, and these properties are highly correlated with each other. It has been found that a higher concentration of each extract will result in greater inhibition of albumin glycation. Thus, it has been proven that the antioxidant capacity of plants and the ability to antiglycate are due to the concentration of phenols and flavonoids [172].

## 6. Synergism in the Action of Medicinal Plant Complexes

Plants have been used as therapeutic agents since the beginning of human history [180]. Texts from ancient Sumeria, India, Egypt, China, and other countries contain recipes using medicinal plants for the treatment of diseases [158]. Today, the use of medicinal plants is still common, with a significant portion of the world’s population relying on herbal natural products and supplements as their primary source of health care [181]. Nearly 20% of adults and 5% of children in the United States use herbal supplements for disease treatment [182]. Despite being used for centuries, the effects of herbal medicines have only been partially studied, and for most natural products on the market, there is no information on which components are responsible for the alleged biological activity. The scientific study of plant-based natural products is challenging due to their enormous complexity and diversity [183]. Efforts in natural product chemistry are generally focused on reducing complexity and identifying individual active components for drug development. However, given that plant complexes, rather than single molecules, are often used for medicinal purposes, interactions between components can be of great importance.

Understanding how combinations of plants and their complexes work together to achieve a specific biological effect can aid in dealing with the ever-increasing threat of disease resistance. Indeed, many diseases are not regulated by a single molecular target but often have a multifactorial causal relationship [184]. Numerous studies have shown that disease resistance is less likely to occur with a combination of compounds than with single active ingredients [185]. Over millennia, plants have evolved to address the multifactorial nature of disease pathogenesis by targeting pathogens through the combined action of structurally and functionally diverse components [180]. Thus, complex mixtures of natural plant substances represent an important resource for drug development, for future success in natural product research, and for understanding the interactions within and between components of mixtures of natural substances. Pharmacological studies of combined effects can be studied at the level of molecular targets, disease pathways, cellular processes, and patient responses [186]. Thus, in vitro, in vivo, preclinical, and clinical studies can provide valuable information about combined effects. Despite the fact that there is a lot of research in this area, these reviews focus on the methodology for interpreting combined effects using molecular and cellular methods [187].

Plant extracts can contain hundreds or even thousands of individual components in varying amounts [188], and the identification of compounds responsible for a given biological effect is a serious problem. Too often, it is assumed that the behavior of a mixture can be described by the presence of only a few known components. However, a number of studies have shown that the overall activity of plant extracts can result from mixtures of compounds with synergistic, additive, or antagonistic activity [189], and often efforts to isolate individual compounds fail because the activity is lost upon fractionation [190]. There are many possible explanations for this problem (including the irreversible adsorption of compounds on the packing of a chromatographic column) [191]. The loss of activity in some cases is caused by the fact that several components are required to observe the biological effect. Many researchers recognize the multifactorial nature of herbal medicines. However, the research methodology applied to herbal mixtures in most cases still tends to either take a reductionist approach (focusing on only one or two “marker compounds”) or completely ignores the issue of chemical composition, testing the biological effects of complex mixtures and complexes with unknown active ingredients. The problem with the latter is that the results tend to be difficult to interpret and reproduce. Many reviews describe the methodologies that currently exist for understanding combined effects in plant complexes.

To successfully generate useful data for understanding the effects of a combination in complex mixtures, one must first select an appropriate biological assay to test the combination. Since the combined effects can manifest through a myriad of mechanisms (including changes in absorption and metabolism, effects on multiple target cells, etc.), in vivo model systems provide the most complete assessment of the overall effect on a living organism [192]. The development of high-throughput in vivo testing of plant complexes and mixtures shows promising possibilities for the identification of multi-target components in mixtures [193]. Despite this, it remains a challenge to solve the complexity of the in vivo systems that require the sacrifice of experimental animals and the maintenance of animal housing. In addition, the results cannot be successfully transferred from one animal model to another. Even when evaluating drug efficacy in human patients, there is often intercellular variability and variability in drug response across patients [158]. Because of this, it is possible that patients receiving combination therapy exhibit increased treatment efficacy because their disease is sensitive to at least one of the drugs in the combination (i.e., independent drug action) rather than due to true combination effects [158].

To overcome some of these problems, a considerable number of researchers work only with in vitro systems. However, many cell-free high-throughput assays that seek molecular targets do not accurately model the biology of an intact cell, making it impossible to discover relevant combinational effects [194]. Thus, it is better to use cellular assays that strike a balance between efficiency and preservation of molecular pathway interactions [195]. Some of the useful cell systems for detecting combined effects in vitro have been discussed in a recent publication by Pemovska et al. [196]. Cellular metabolism is a dynamic network of regulated pathways that is often reprogrammed during cancer and aging, and is recognized as a new key area of study. The first observations that transformed cells exhibit a distinct metabolic program were made by Otto Warburg almost a hundred years ago. The Warburg effect describes the phenomenon of cancer cells predominantly undergoing glycolysis and the conversion of carbon to lactate even under conditions of high oxygen content [197]. In cancer and aging, genetic events activate signaling pathways that subsequently modulate cellular metabolism to meet increased bioenergetic, biosynthetic, and redox needs [198]. Moreover, it contributes to the initiation and progression of cancer and aging, and is usually accompanied by changes in the expression of metabolic enzymes and transporters, which are important for the absorption and distribution of nutrients along biomass formation pathways, which ultimately affects the response to therapy [199,200]. Therefore, the metabolic changes specific to cancer and aging provide not only a selective advantage for survival, but also introduce metabolic limitations that provide a unique opportunity for therapeutic targeting [201]. In addition to selecting appropriate cell systems for biological testing, it is important to mimic physiological conditions in the assay itself. Indeed, most of the media used to grow cells for biological testing do not mimic physiological conditions, affecting the metabolism and phenotypic response of the cells under study [202]. Similarly, the conditions of biological assays can lead to dynamic residual complexity where the sample undergoes chemical change caused by the environment, making it difficult to interpret the results [203]. In their recent publication, Vande Voorde et al. [202] illustrated that the use of a complex culture medium designed to mimic the physiological environment of cancer cells prevents the formation of undesirable phenotypic artifacts and improves the transferability between in vitro assay results and in vivo tumor models. The use of physiologically relevant media also increases the likelihood that the components that elicit a biological response during biological testing will be soluble and stable in the biological system, facilitating the identification of active components. Primary tissue assays composed of multiple cell types, such as those used to screen drug combinations for anti-inflammatory activity in mixed lymphocyte cultures, can also be used to identify combination effects that work through multitarget mechanisms [197]. However, when screening for biological activity in vitro, investigators should be aware of potential false-positive results arising from interference compounds commonly referred to as pain, which are often identified as hits in biological screenings [203]. These false-positive results can be generated due to multiple mechanisms, including fluorescence quenching, aggregation effects, chemical reactivity, oxidation/reduction, membrane disruption, and residual complexity [203]. Synergistic results are often found in aqueous media due to aggregation effects, which can be minimized by adding a detergent to the media [204].

In addition to careful selection of the biological system to study the effects of the combination, it is necessary to collect data to effectively compare the combination of drugs with extracts and individual substances [190]. Combined effects, including synergism and antagonism, can manifest themselves over a wide range of concentrations, so it is necessary to test different ratios of the studied samples [205]. A study [206] found that human serum is a vital component of the host’s innate immunity that acts as the first line of defense against invading pathogens. A key player in serum-mediated innate immune defense is a system of more than 35 proteins, collectively referred to as the complement system. Upon pathogen exposure, these proteins are activated in a cascade manner, eventually forming a membrane attack complex (MAC) on the surface of the pathogen, which directly lyses the bacterial cell. MAC formation had been demonstrated in vitro using a serum bactericidal assay (SBA) that works in the absence of blood cellular components after serum has been incubated with bacteria. The age-related differences in the bactericidal activity of human blood serum against *Pseudomonas aeruginosa* have been described. It has been demonstrated that sera from young adults were highly effective in killing *Pseudomonas aeruginosa* in vitro compared to children and the elderly. The sera of the elderly were severely compromised when killing *P. aeruginosa*, while the sera of young people showed an increased level of killing. The data revealed a positive correlation between age and bacterial cell death with higher coefficients of determination of 0.34, 0.27 and 0.58 after 60, 90 and 120 min of incubation, respectively. Therefore, this study highlighted age-related differences in the bactericidal activity of human sera [207]. One of the simplest methods for identifying potential combined effects is to test samples individually and in combination, and to determine if the combined effect of the samples is greater than, equal to, or less than the expected sum of the two samples individually.

In addition to concentration-based approaches to evaluating combined effects, time-based approaches have been developed and applied to determine antimicrobial synergism and describe the relationship between bactericidal activity and sample concentration [208]. This method involves exposing a chosen pathogen to an inhibitor (or combination of inhibitors), sampling the cultures at regular intervals, serially diluting and incubating aliquots, and comparing the resulting colony-forming units. The resulting dose–response curve can be used to determine additive, synergistic, and antagonistic effects [209]. Synergism can occur through a variety of mechanisms, including pharmacodynamic synergy through multiple target effects, pharmacokinetic synergy through modulation of drug transport, penetration and bioavailability, elimination of side effects, and the manifestation of disease resistance mechanisms [209]. While the general mechanisms by which synergistic effects may occur are relatively well understood, the mechanisms by which specific herbal preparations exert synergistic effects remain largely unknown [210], hindering attempts to standardize and optimize them for therapeutic purposes. Only by understanding the nature of the synergistic activity of plant extracts is it possible to optimize safe and effective drugs for the treatment of diseases.

Cancer cells and pathogens can quickly become resistant to drugs containing a single compound, and many cancers and resistant bacterial infections are treated with complex multi-target drug combinations to overcome the development of resistance [211]. Plants have long had to defend themselves against multifactorial diseases and have evolved to produce a variety of active components that can adhere to cell membranes, intercalate into RNA or DNA, and bind to numerous proteins [212]. Pharmacodynamic synergy results from targeting multiple pathways that may include enzymes, substrates, metabolites, ion channels, ribosomes, and signaling cascades [210]. Pharmacodynamic synergism may occur through complementary actions in which synergists in a mixture interact with multiple points in the pathway, leading to up-regulation of the drug-targeting process or down-regulation of competing mechanisms. By selectively altering target activity and expression through complementary actions, pharmacodynamic synergists can both enhance the beneficial effects of treatment and reduce the side effects of the disease [211]. For example, numerous studies have shown that many plants have synergistic neuroprotective effects both in vivo and in vitro by inhibiting free radical formation, scavenging reactive oxygen species, regulating mitochondrial target gene expression, and reducing overstimulation of nerve cells by neurotransmitters [213]. 

## 7. Conclusions

A significant increase in the proportion of the elderly population in developed countries is accompanied by an increase in mortality from major diseases of old age (diseases of the cardiovascular system, malignant neoplasms, neurodegenerative processes, reduced resistance to infection, and diabetes mellitus). One of the promising objects for the prevention of antioxidant, anti-inflammatory, neuroprotective, and anti-glycation aging processes are the components of medicinal plants.

Millions of people in the world suffer from chronic neurodegenerative diseases (Parkinson’s and Alzheimer’s diseases, Huntington’s chorea, hyperprolactinemia, etc.), which, despite therapy, end in disability and/or death. Medicinal plants, due to the presence of biologically active substances, can play an important role in the prevention of the development of neurodegenerative diseases such as Alzheimer’s disease. The mechanisms of this effect are not always known. Perhaps, antioxidant and adaptogenic mechanisms play a role here.

The search, methods of isolation, and study of promising natural sources of substances with antiradical and antioxidant activity are currently one of the urgent tasks for modern medicine, pharmacy, cosmetology, and the food industry to reduce the effects of aging of the human body. Medicinal plants play an important role in the development of new potent anti-inflammatory drugs through the production of secondary metabolites with biological activity. Medicinal plants are used in place of non-steroidal anti-inflammatory drugs, given that the use of these drugs is associated with several side effects, among which are unwanted effects on the gastrointestinal tract and kidneys.

The glycation process, which is enhanced by hyperglycemia, underlies the pathogenesis of micro and macrovascular complications of diabetes mellitus (DM), which is common in the elderly. It was found that the antiglycation properties of herbal extracts and their complexes strongly correlated with their antioxidant capacity (trapping DPPH-radicals). Like antioxidant capacity, anti-glycation activity correlated strongly with phenol and flavonoid content.

In recent years, the concept of synergy in mixtures of natural plant substances has attracted attention, and the importance of multipurpose combination therapy in human aging has come to the fore. However, the classification of combined effects in complex mixtures and the identification of constituents remains a challenge, especially when most known tools have been developed to reduce the complexity of mixtures of natural substances. Furthermore, there is still disagreement in this field about which reference models are best for identifying combined effects, making it difficult to interpret studies. The metabolomic and biochemometric approaches are promising tools for studying synergy and have only just begun to be used to identify the components involved in the combined effects [197].

## Figures and Tables

**Table 1:** Anti-neurodegenerative action of various plant components.

Plant	Active Components	Activity	Sources
*Citrus limon*	Nobiletin, flavonoids	Neuroprotective action	[76]
	Tangeretin	Neuroprotective action	[76]
	Essential oil Glycosides Phytoncides Macro-, microelements Organic acids Vitamins Pectin substances	Therapeutic potential in inflammatory and degenerative processes in the nervous tissue accompanied by microglia activation	[115]
	Narirutin	Therapeutic effect in Alzheimer’s disease	[115]
	Naringenin	Prevention of impaired dopamine synthesis in the brain and the development of Parkinson’s disease	[76]
*Rauvólfia serpentína*	Reserpine Macro-, microelements Organic acids Vitamins Indole flavonoids	Therapy of hypertension and psychotic disorders (schizophrenia, anxiety, insomnia); reduces Aβ toxicity in an Alzheimer’s disease model	[126]
*Punica granatum*	Punicalagin Macro-, microelements Organic acids	Amyloid load reduction and behavior improvement in an Alzheimer’s model	[127]
	Vitamins Flavonoids Fatty acids	β-secretase inhibition	[128]
	Ellagic acid	β-secretase inhibition	[128]
*Terminalia chebula*	Chebulic acid, ellagic acid Macro-, microelements Vitamins Triterpenoids Saponins Quinolinic acid	Neuroprotective action	[77]
*Sílybum mariánum*	Buformin Flavonoids	Immunosuppressive action preventing memory impairment	[79]
	Silibin Silychristin Silydianin	Immunosuppressive action preventing the destruction of nerve cells caused by oxidation	
*Inula britannica*	Rosmarinic acid Flavonoids Essential oil Carotene Lactones Tannins	Anticholinesterase activity	[87]
*Fumaria officinalis*	Alkaloids Glycosides Vitamins Organic acids Sugars Resins Essential oil traces	Anticholinesterase activity	[90]
*Vitis vinifera*	Gallic acid Organic acids	Prevention of formation and accumulation of amyloid fibrils	[129]
	Limonene Geranyl Phenyl acetate Squalene	Increasing the rate of transport of β-amyloid into the blood	[130]
*Curcuma longa*	Curcumin Carbohydrates Essential oils Fatty acids Curcuminoids	Prevention of toxic-induced degradation of black dopaminergic neurons and impeding the development of Parkinson’s disease	[131]
*Acorus calamus*	β-azarone Essential oils Polysaccharides phenolic compounds	Inhibition of the release of pro-inflammatory mediators and cytokines; decreased JNK phosphorylation, inhibition of NF-κB nuclear translocation	[106]
*Filipendula ulmaria*	Kaempferol, luteolin, apigenin	Therapeutic effect in neurodegenerative diseases	[122]
	Nitrogen compounds Higher fatty acids Ethers	Prevention of demyelination processes in encephalomyelitis	[123]
*Viscum album*	Glabridin Flavonoids Terpenoids Hormones	Protection against deterioration of cognitive processes and memory caused by exposure to chemical agents	[132]

## Data Availability

The data are included in the manuscript.
